# S09-2 The impact of mass-media campaigns on physical activity: a review of reviews through a policy lens

**DOI:** 10.1093/eurpub/ckac093.046

**Published:** 2022-08-29

**Authors:** Nicolette R den Braver, Enrique Garcia Bengoechea, Sven Messing, Liam Kelly, Linda J Schoonmade, Kevin Volf, Joanna Zukowska, Peter Gelius, Sarah Forberger, Catherine Woods, J Lakerveld, on behalf of the Policy Evaluation Network (PEN) Consortium

**Affiliations:** 1 Department of Epidemiology & Data Science, Amsterdam Public Health Research Institutes, Amsterdam University Medical Centres, De Boelelaan 1089a, Amsterdam 1081 HV, The Netherlands; 2 Upstream Team, www.upstreamteam.nl, De Boelelaan 1089a, Amsterdam 1081 HV, The Netherlands; 3 Physical Activity for Health Research Cluster, Health Research Institute, Department of Physical Education and Sport Sciences, University of Limerick, Limerick, Ireland; 4 Research & Innovation Unit, Sport Ireland, Ireland; 5 Department of Sport Science and Sport, Friedrich-Alexander-Universität Erlangen-Nürnberg, Gebbertstr. 123b, 91058 Erlangen, Germany; 6 Medical Library, Vrije Universiteit Amsterdam, De Boelelaan 1117, P.O. Box 7057, 1007 MB Amsterdam, The Netherlands; 7 Faculty of Civil and Environmental Engineering, Gdansk University of Technology, Narutowicza 11, Gdansk 80-233, Poland; 8 Leibniz Institute for Prevention Research and Epidemiology – BIPS, 28359 Bremen, Germany

**Keywords:** physical activity, benchmarking, policy, implementation, sport, transport, mass media

## Abstract

**Background:**

This review of reviews aims to summarize the evidence from published reviews on the effectiveness of mass-media campaigns to promote physical activity (PA), or PA-related determinants, and identify policy-relevant recommendations related to successful PA campaigns.

**Methods:**

An extensive literature search was performed on March 1st, 2021. Reviews that evaluated the impact of campaigns on distal (e.g., PA) and/or proximal outcomes of PA (awareness, knowledge etc.) and that targeted the general population or subsets were included. Quality of reviews was assessed using the AMSTAR-2 tool. Policy-relevant recommendations were systematically derived and synthesized, and formulated as good practice statements. A protocol was registered beforehand (ID: CRD42021249184).

**Results:**

A total of 1,915 studies were identified, of which 22 reviews were included. Results indicate that the most consistent evidence was found for the effectiveness of mass-media campaigns on proximal outcomes, while the evidence for distal outcomes was mixed. Good practice statements were derived: 1) to achieve behaviour change, mass-media is an important component of larger, multilevel, and multicomponent strategies, 2) mass-media strategies should be coordinated and aligned at local- and national-level, and be sustained, monitored and resourced at these levels, 3) media should be tailored to reduce socioeconomic inequalities.

**Conclusions:**

Mass-media can play an important role in the promotion of PA. In general, evidence was more inconsistent for effectiveness on distal outcomes than for proximal outcomes. The policy-relevant recommendations identified will serve to inform the PA environment policy index (PA-EPI), a tool for monitoring, evaluating and benchmarking government progress in implementing public policies.

